# Polyurethane scaffolds seeded with CD34^+^ cells maintain early stem cells whilst also facilitating prolonged egress of haematopoietic progenitors

**DOI:** 10.1038/srep32149

**Published:** 2016-08-30

**Authors:** Charlotte E. Severn, Hugo Macedo, Mark J. Eagle, Paul Rooney, Athanasios Mantalaris, Ashley M. Toye

**Affiliations:** 1 School of Biochemistry, Biomedical Sciences Building, University of Bristol, Bristol, BS8 1TD, United Kingdom; 2 National Institute for Health Research Blood and Transplant Research Unit (NIHR BTRU) in Red Blood Cell Products, University of Bristol, Bristol, BS8 1TD, United Kingdom; 3 Biological Systems Engineering Laboratory, Department of Chemical Engineering, Imperial College London, London, SW7 2AZ, United Kingdom; 4 Tissue and Eye Services, NHS Blood and Transplant, Liverpool, L24 8RB, United Kingdom

## Abstract

We describe a 3D erythroid culture system that utilises a porous polyurethane (PU) scaffold to mimic the compartmentalisation found in the bone marrow. PU scaffolds seeded with peripheral blood CD34^+^ cells exhibit a remarkable reproducibility of egress, with an increased output when directly compared to human bone scaffolds over 28 days. Immunofluorescence demonstrated the persistence of CD34^+^ cells within the scaffolds for the entirety of the culture. To characterise scaffold outputs, we designed a flow cytometry panel that utilises surface marker expression observed in standard 2D erythroid and megakaryocyte cultures. This showed that the egress population is comprised of haematopoietic progenitor cells (CD36^+^GPA^−/low^). Control cultures conducted in parallel but in the absence of a scaffold were also generally maintained for the longevity of the culture albeit with a higher level of cell death. The harvested scaffold egress can also be expanded and differentiated to the reticulocyte stage. In summary, PU scaffolds can behave as a subtractive compartmentalised culture system retaining and allowing maintenance of the seeded “CD34^+^ cell” population despite this population decreasing in amount as the culture progresses, whilst also facilitating egress of increasingly differentiated cells.

The human body efficiently compartmentalises the red blood cell manufacturing process in the bone marrow, producing 2.5 million reticulocytes per second for an entire lifetime using only a tiny contingent of haematopoietic stem cells (HSC). The HSCs in the bone marrow reside within the endosteal niche where they undergo symmetric and asymmetric division^1^,^2^,^3^,^4^,^5^. HSCs differentiate to first a multipotent progenitor (MPP) and then a common myeloid progenitor (CMP) most often characterised as CD34^+^CD38^+ ^6,7,8. Once restriction to the megakaryocyte/erythroid progenitor (MEP) stage occurs cells become; CD34^+^/GPA^+ ^9, CD34^+^/CD38^low/+ ^10, CD41^+^/GPA^+ ^11 and more recently CD34^+^ cells were shown to progress from CD34^+^/CD36^−^ as a CMP and then CD34^+^/CD36^+^MEPs^12^,^13^. However there is now evidence that true CMP populations are a rare component of the haematopoietic tree and instead bipotent cells are able to differentiate down the erythroid and megakaryocyte lineages or the myeloid and megakaryocyte lineages that arise directly from an MPP^14^,^15^. Upon lineage commitment cells express lineage specific markers such as GPA and band 3 for erythroid cells and CD42b and CD61 in the megakaryocyte lineage^16^,^17^,^18^,^19^. Lineage differentiation is dependant upon cytokines, namely erythropoietin (EPO) for erythroid development and thrombopoietin (TPO) for the generation of megakaryocytes and their progenitors, although TPO is also known to influence HSCs^20^,^21^,^22^,^23^,^24^,^25^,^26^.

Successful protocols have been generated to produce reticulocytes using HSCs isolated from adult peripheral blood^27^,^28^,^29^,^30^,^31^,^32^, umbilical cord blood^32^,^33^,^34^,^35^ and embryonic stem cells^36^,^37^; although with varying yields of reticulocytes. Proof of principle has also been provided for the safety of cultured RBC (cRBC), as 2.5 ml of packed reticulocytes generated *ex vivo* were transfused into a single volunteer^30^. More recently 5 ml packed reticulocytes have been manufactured but further scale-up is required to reach an adult therapeutic dose^31^; these initial successes were achieved using static flasks or stirrer flasks^30^,^31^. The challenge going forward for cRBC production is that the current culture conditions cause HSCs to be rapidly pushed into erythroid lineage commitment, eventually exhausting the initial stem cell pool and limiting expansion capacity. Furthermore, high-density culture is difficult due to the increased likelihood of spontaneous terminal differentiation and so vast culture volumes are needed (reviewed in ref. 38 and 39).

One option is better recapitulation of the bone marrow structure and microenvironment to increase yields and longevity of erythroid cultures. Multiple research groups have attempted to recreate the honeycomb like architecture of the human bone marrow using three-dimensional scaffold culture systems with the ultimate aim of reproducing the whole of erythropoiesis within the scaffold environment. At present there is no consensus as to the optimal scaffold material, culture conditions or cell type to use for seeding, making direct comparisons between studies difficult. One approach is to seed HSCs directly onto scaffolds with a number of materials already investigated including the biocompatible PU used here^40^, hydrogels^41^, fibrin^42^, bio-derived bone^43^, PET^44^, and non-woven polyester disks^45^.

In this study we compare the output from a highly porous PU scaffold seeded with CD34^+^ cells to that produced from a de-cellularised human bone derived scaffold, with the aim of demonstrating compartmentalisation of early stem cells in the honeycomb structure. We describe techniques that assess the impact of changes on either scaffold occupancy or in scaffold egress following an alteration in culture conditions. Finally we demonstrate that static PU scaffold cultures offer the opportunity to harvest haematopoietic progenitors across a longer time period compared to traditional 2D cultures.

## Results

### Haematopoietic progenitors continuously egress from CD34^+^ seeded polyurethane scaffolds over 28 days of culture

**Figure 1:**
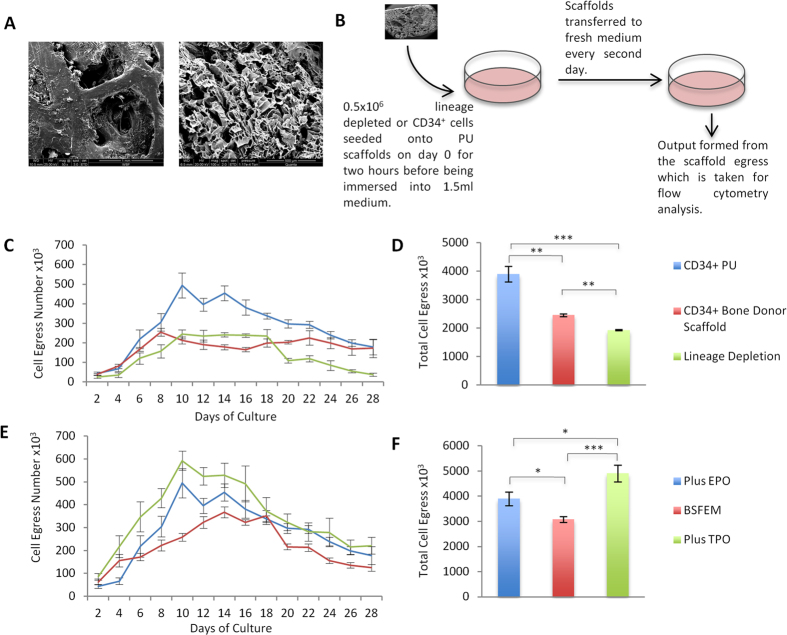
Polyurethane and bone donor scaffolds sustain cellular egress for a 28-day period. (**A**) Scanning electron micrographs (SEM) of the human bone donor (left) and polyurethane (PU) (right) scaffolds, scale bars are 1 mm and 500 μm respectively. (**B**) Schematic of the scaffold culture protocol. Scaffolds are seeded with 0.5 × 10^6^ lineage depleted or CD34^+^ cells on day 0 in 100 μL of BSFEM for 2 hours before being immersed in 1.5 mL of medium. Scaffolds are transferred to a fresh well of medium every 2 days and the output is formed from the previous well. The process continues for the 28 day culture period. (**C**) Cell counts of egress from PU scaffolds seeded with CD34^+^ cells (blue), bone donor scaffolds seeded with CD34^+^ cells (red) and PU scaffolds seeded with lineage depleted cells (green) cultured in BSFEM with the addition of EPO, across the 28 day culture period. (**D**) Cumulative egress from each scaffold in (**C**) over the entire 28 day culture period (**E**) Cell counts of egress from PU scaffolds seeded with CD34^+^ cells in BSFEM (red) or with EPO (blue) or TPO (green) across the 28 day culture period. (**F**) Cumulative egress from each scaffolds in (**E**) over the entire 28 day culture period. Outputs for the CD34^+^ plus EPO experiments in graphs (**C–F)** are derived from a single set of experiments. N = 3 independent experiments in at least duplicate for PU scaffolds, N = 2 in triplicate for human bone donor scaffolds. Error bars represent the standard error of the mean. Significance was tested using students T-test, *P ≤ 0.05, **P ≤ 0.01, ***P ≤ 0.001.

The three-dimensional scaffolds used in this work are 0.175 cm^3^ PU or de-cellularised bone scaffolds, that exhibit a sponge like honeycomb interior for cell culture (Fig. 1A). PU has been previously shown to support expansion of cord blood MNCs^46^. The experimental protocol is outlined in Fig. 1B. A population of 0.5 × 10^6^ lineage depleted peripheral blood mononuclear cells (PBMNCs) or CD34^+^ cells from adult peripheral blood were seeded on day 0 and cultured in StemSpan supplemented with dexamethasone, SCF, IL-3 and IGF (referred to as basic serum free expansion medium, BSFEM) with the addition of 2 U/mL EPO. We hypothesized that the lineage depleted population would contain early CD34^−^ stem cells^28^ and thus may provide a greater diversity of stem cells that could increase the yield of erythroblasts. Cellular egress was measured as the cells left behind in the well after moving the scaffold to fresh media; therefore, unlike previous scaffold cultures where generally the output resided inside the scaffold, the quantifiable output here was taken to be the cells that spontaneously egressed from the scaffold.

Continuous cellular egress from the PU scaffold was observed over a 28 day period (Fig. 1C–F). When PU scaffolds were seeded with CD34^+^ cells, maximum egress occurred on day 10 (4.94 × 10^5^) (Fig. 1C). The lineage depleted population contains a variable proportion of CD34^+^ cells (10–15%, data not shown), and significant reduction in scaffold egress (P < 0.001) occurred when PU scaffolds were seeded with lineage depleted cells compared to seeding with CD34^+^ cells (Fig. 1D). There was continuous egress from PU and human bone scaffolds seeded with CD34^+^ cells although the peak of egress was slightly earlier at day 8 for the bone scaffolds. However it should be pointed out that the total cell egress from the bone scaffold was significantly lower than the PU scaffold culture (P < 0.01), 2.45 × 10^6^ and 3.90 × 10^6^ for the human bone and PU scaffolds respectively (Fig. 1D). Due to difficulties in seeding this more rigid scaffold and the natural variability of pore structure, it is possible that fewer cells were seeded and/or retained than in the PU scaffolds. This was supported by the observed higher egress from the bone scaffold compared to PU at day 2 of culture, which represents cells that had not been retained during seeding alongside egress material, therefore a reduction in expansion capacity would be expected. A de-mineralised and de-cellularised bone scaffold was also tested, however this scaffold did not perform well for this application (data not shown). Therefore this work confirms that the CD34^+^ population is the preferential seeding material for maximal production and reproducibility, and that PU scaffolds are optimal in terms of cellular egress compared to the human bone derived scaffolds.

### The effect of the inclusion of cytokines on CD34^+^ cells in PU scaffold cultures

To determine the effect of cytokines on the output from CD34^+^ PU cultures, scaffolds were cultured in BSFEM alone or BSFEM containing either EPO or TPO, the latter included for its known influence on HSCs expansion. Figure 1E demonstrates that the peak of cellular egress in BSFEM was observed on day 14 (3.67 × 10^5^) and slightly earlier at day 10 for both plus EPO and TPO (4.94 × 10^5^ and 5.92 × 10^5^ respectively). The difference between maintenance in a basic media or addition of cytokines was found to be significant for EPO (P < 0.01) and highly significant with the inclusion of TPO (P < 0.001) (Fig. 1F).

**Figure 2:**
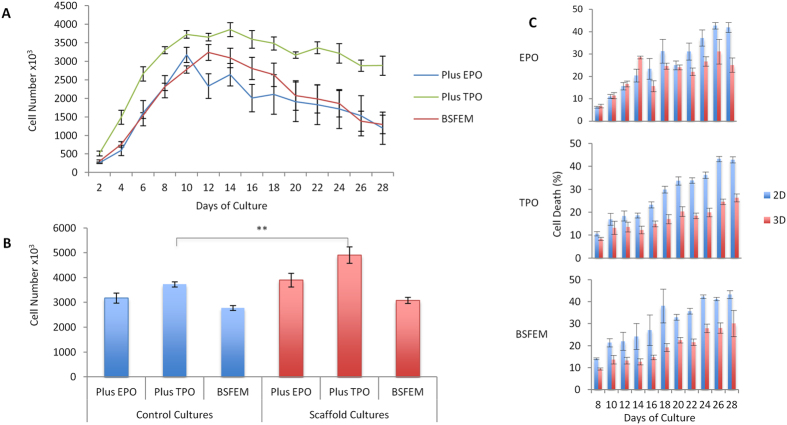
Parallel control cultures grown in the absence of scaffolds are sustained for the duration of the culture period however cell death is increased in controls compared to scaffold cultures. (**A**) Cell counts of high-density control cultures seeded with 0.5 × 10^6^ CD34^+^ cells and maintained in 1.5 mL either BSFEM or with the addition of EPO or TPO. Dead cells were excluded using propidium iodide. (**B**) The average cumulative egress for scaffolds and the maximal point of control cultures, taken as day 12 for BSFEM, +EPO and +TPO. Each culture was seeded with 0.5 × 10^6^ CD34^+^ cells on day 0. The difference between the cumulative scaffold egress and peak of control cultures grown in the absence of scaffolds was found to be statistically significant in the plus TPO condition (P = 0.00557) but not in plus EPO (P = 0.05564) or BSFEM (P = 0.06647). (**C**) Cell death was assessed using propidium iodide as per manufacturers instructions and expressed as percentage cell death across the BSFEM, +EPO and +TPO conditions in both scaffold egress (red) and control cultures grown in the absence of scaffolds (blue), every second day for the 28 day period. Cell death was found to be statistically significant between control and scaffold cultures in BSFEM and upon the addition of TPO (P = ≤ 0.05). N = 3 independent experiments in at least duplicate for PU scaffolds, error bars represent the standard error of the mean and significance was tested using the students T-test.

Alongside 3D scaffold cultures, parallel control cultures of 0.5 × 10^6^ CD34^+^ cells maintained in 1.5 mL media minus a scaffold were set up to compare cell expansion and differentiation in the different culture conditions. To mimic the subtractive nature of scaffolds, the average number of cells that egressed from the scaffold culture was removed from the control culture. To mimic the transfer of scaffolds to a new well with fresh medium, full medium changes were performed for control cultures. Surprisingly, a large number of the control cultures also persisted for up to 28 days under all culture conditions tested despite reaching a high-density (Fig. 2A). A limitation of removing the average number of cells however is that material is being removed at random and all cells are in the same pool unlike the scaffold which is effectively a subtractive culture; therefore a greater number of CD34^+^ cells may be removed from one culture compared to the next thus causing a reduction in proliferation which leads to the early exhaustion of cultures. This effect is most evident in several BFSEM alone cultures and in cultures with EPO where the entire population of cells are exhausted or undergo apoptosis (Supplemental Figure S2). The exhaustion of these cultures may therefore be a result of the culture conditions not supporting the terminal differentiation of the cells into the erythroid lineage. The average culture progression for each of the three conditions in the control cultures reveals a peak at day 10 for plus EPO (3.18 × 10^6^), day 12 for BSFEM (3.24 × 10^6^) and day 14 for plus TPO (3.86 × 10^6^) (Fig. 2A). The peak of control cultures (taken as day 12 for all conditions) is the highest production point reached of the control cultures, cells then begin to spontaneously differentiate and die. Therefore this peak population point was chosen to compare to the cumulative total cell egress from scaffold cultures, which revealed there is only a significant difference (P = < 0.01) with the inclusion of TPO (Fig. 2B).

To investigate whether scaffolds are beneficial to the survival of cultured cells, cell death was assessed in both the scaffold and control cultures. Cell death was increased in the controls compared with the scaffold cultures, results which were largely unaffected by the presence or absence of cytokines. This increase was found to be statistically significant when assessed across the whole culture period, when cultures were maintained in either BSFEM or with the addition of TPO (P = < 0.05, Fig. 2C), thus suggesting that the honeycomb structure of the PU scaffold may aid in the survival of developing cells.

### Surface marker expression in standard 2D erythroid and megakaryocyte cultures

Surface marker expression in 2D erythroid and megakaryocyte cultures was characterised using flow cytometry in order to generate a developmental profile under defined culture conditions. The markers used were: CD34 to identify haematopoietic stem and progenitor cells, CD36 which is expressed during the bifurcation point from CMP to MEP^12^,^13^, CD61 which is expressed on MEPs, megakaryocyte progenitors, megakaryocytes and platelets, GPA which is expressed on erythroblasts and reticulocytes and CD14 to identify monocytes and macrophages.

**Figure 3:**
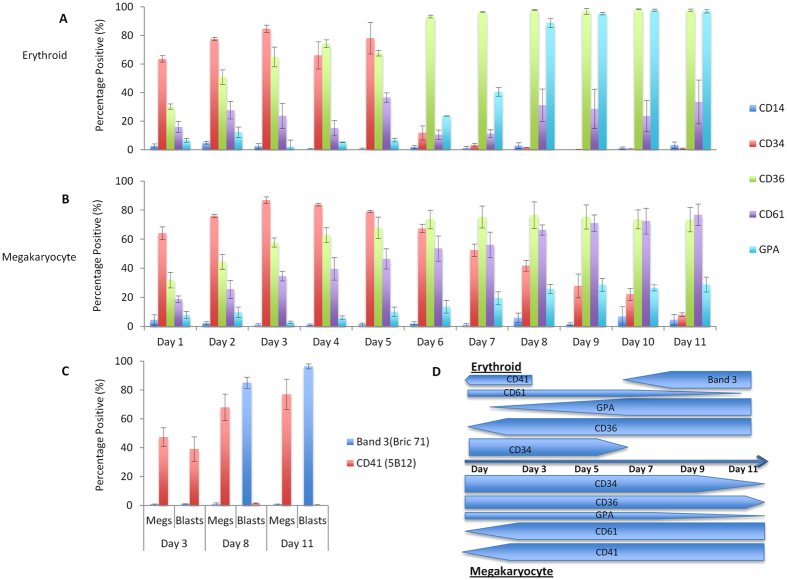
Cell surface expression during standard erythroid and megakaryocyte 2D cultures. (**A,B**) Immunophenotype of cells analysed daily from standard 2D erythroid and megakaryocyte cultures expressed as a percentage of the live cell population for each of the following cell surface markers; GPA^+^ (APC), CD61^+^ (APC Vio770) and CD34^+^ (VioBlue). Erythroid cultures are first cultured in expansion medium and then differentiation medium from day 7, whereas megakaryocyte cultures remain in the same megakaryocyte culture medium throughout. (**A**) Erythroid culture, (**B**) megakaryocyte culture. (**C**) Expression of mature surface markers band 3 (Bric71) and CD41 (5B12) in both erythroid (blasts) and megakaryocyte (megs) cultures on days 3, 8 and 11 of culture; monoclonal primary antibodies were coupled to an APC secondary antibody. Expression is shown as a percentage of the total live cell population. N = 3 independent experiments with error bars representing the standard error of the mean. (**D**) Daily analysis of megakaryocyte and erythroid cultures as assessed with flow cytometry for 11 days with the following antibodies; CD34, CD36, CD61, CD14 and GPA. Above erythroid, below megakaryocyte culture. Schematic is a representation of the duration of expression in addition to the level of expression for each cell surface marker. N = 3 independent experiments.

Erythroid and megakaryocyte cultures were observed to display similar cell surface marker profiles during the first 5 days of culture, with expression of CD34 reducing, CD61 and GPA low and CD36 increasing (Fig. 3A,B); thus providing evidence that cells are initially not restricted to either the erythroid or megakaryocyte lineage. From day 6 in culture, GPA expression increases in erythroid cultures. In megakaryocyte cultures CD61 increases from day 1, whereas in erythroid cultures CD61 expression remains low throughout. The schematic in Fig. 3D provides an overview of erythroid and megakaryocyte markers during standard 2D culture. It is important to note that, despite detectable expression of GPA and CD61 in both conditions, the cell surface density (MFI) of GPA in megakaryocyte cultures and CD61 in erythroid cultures is very low (Supplemental Figures S1 and S3). By day 11 mature cell surface markers are expressed including band 3 for erythroid and CD41 for megakaryocyte cultures showing the initiation of lineage-specific differentiation (Fig. 3C).

### Cells that egress from PU scaffolds exhibit an erythroid progenitor phenotype

**Figure 4:**
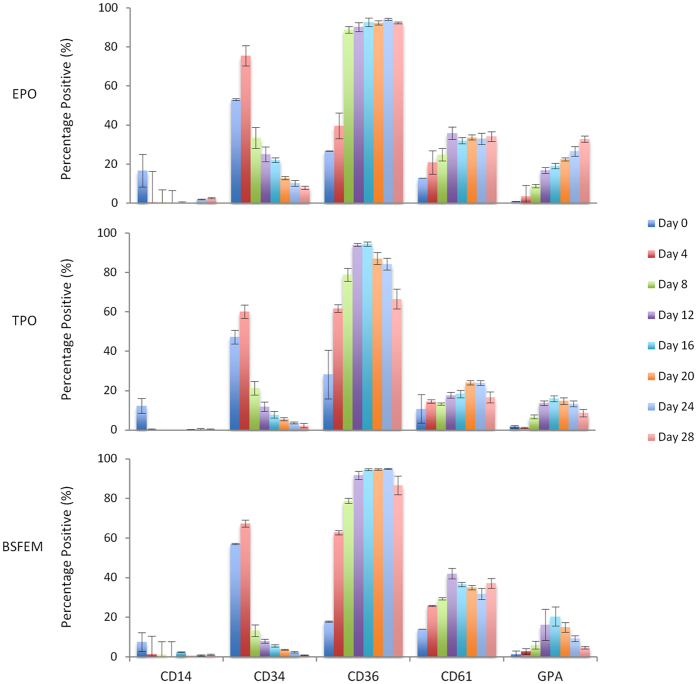
Flow cytometry analysis reveals the cells that egress from the scaffolds are largely early erythroid progenitors. Cell populations that egress from scaffold cultures were assessed every 4 days using antibodies to detect the following populations; CD14 (FITC), CD34 (VioBlue), CD36 (PE), CD61 (APC-Vio770) and GPA (APC). Cellular populations are shown as a percentage of the total live cell population for each condition, plus EPO, plus TPO and BSFEM. N = 3 independent experiments in at least duplicate, error bars represent the standard error of the mean.

The flow cytometry panel described above was applied to scaffold egress throughout the culture period. A high proportion of the cells egressed from PU scaffolds seeded with CD34^+^ cells are early erythroid progenitors as indicated by high CD36 and low GPA; although as expected GPA expression was slightly higher upon the addition of EPO (Fig. 4 and Supplemental Figure S4). Importantly, a small but detectable population of CD34^+^ cells (5.6%, 7.8% and 22% in BSFEM plus TPO and plus EPO scaffold cultures respectively at day 16) persists throughout the 28 day culture period (Fig. 4). CD34 expression levels reduce as the culture progresses; for example on day 4, MFI is 391 and decreases to 39 by day 16 when scaffolds are cultured in BSFEM (Supplemental Figure S4). Therefore this prolonged egress of early progenitors provides evidence for the retention of input material but also delayed maturation within PU scaffold cultures. CD61 is expressed consistently on 20–30% of the egress population under all conditions tested (Fig. 4). The CD34/CD36 expression profile provides further evidence for a persistence of MEPs for the duration of the culture with a clear transition of CD34^+^/CD36^−^ CMP population in the first week of culture to a CD34^+^/CD36^+^ MEP population for the remaining 3 week culture period (Supplemental Figure S5)^12^,^13^. Expression levels for both GPA and CD61 were consistently low on egressed cells, corresponding to the surface marker expression observed on early progenitors in the early stages of standard 2D erythroid and megakaryocyte cultures (Supplemental Figure S4). Only a small number of CD14 positive cells were detected, however expression was variable across cultures, as illustrated by large error bars (Fig. 4).

Surface expression was found to be strikingly similar between the scaffold egress and parallel control cultures (that are grown under the same conditions but lack scaffolds) in terms of both levels (MFI) and percentage of population (Supplemental Figures S3 and S4). This is consistent with the fact that the scaffold and control cultures were seeded with the same CD34^+^ starting material and indicates that these cultures are generating similar haematopoietic progenitors during the culture’s lifetime. Expression of CD34^+^ was however, found to be increased in control cultures over scaffold egress, suggesting retention within the scaffold structure (Fig. 4 and Supplemental Figure S3).

### 3D cultures enable persistence of CD34^+^ cells within PU scaffolds

**Figure 5:**
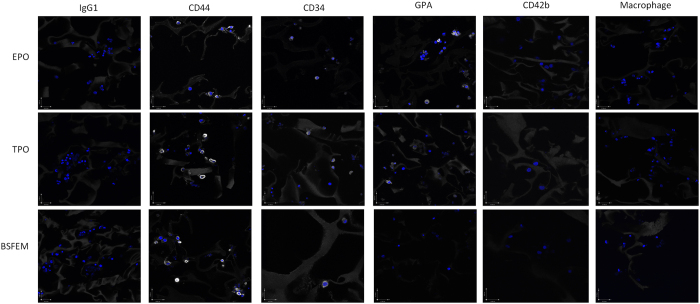
CD34^+^ cells persist to the culture endpoint within the honeycomb structure of PU scaffolds. Polyurethane scaffolds were cultured for 28 days and paraffin wax blocks cut to 10 μm sections. Scaffold sections were probed with CD44 (Bric 235), CD34 (Birma K3), GPA (Bric 256), CD42b (BioLegend), and a macrophage marker (calprotectin) and coupled to Alexa 647 secondary antibody (white), DAPI was used to stain the nucleus (blue). Images were acquired using a Leica SP5-AOBS confocal laser scanning microscope attached to a Leica DM I6000 inverted epifluorescence microscope using the 60x lens (N.A. 1.4), scale bars are 27 μm, images are representative and N = 3.

To determine whether an internal bone marrow “niche-like” environment is established within PU scaffold cultures, cells residing within the scaffold at the end point of culture, day 28 were visualised using histology techniques (see materials and methods). DAPI was used to stain the nucleus but also demonstrates distribution throughout the scaffold (Fig. 5). CD44 a marker known to be present on many haematopoietic lineages including the erythroid lineage^47^ was used to identify a large proportion of cells in scaffold sections from all three conditions (Fig. 5). Notably there was a persistence of CD34^+^ cells, consolidating the flow cytometry work above and strengthening the argument for retention of CD34^+^ material and delayed maturation (Fig. 5). In addition, expression of GPA was detected in both the plus EPO and TPO cultures, albeit it at a higher level in the plus EPO cultures; however there is very little or no expression in the BSFEM cultures (Fig. 5). There is also no evidence of megakaryocytes when using CD42b (Fig. 5). Taken together, one explanation may be that the CD34^+^ cells residing within scaffolds cultured without added cytokines are retained in a more immature state, with a proportion of cells exhibiting detectable CD34^+^ expression after 28 days of culture in each condition. Finally, detection of mature macrophages was attempted using L1 calprotectin marker but no positive cells were detected (Fig. 5), indicating a “niche-like” environment exhibiting erythroblastic islands does not develop within the scaffold cultures.

### Scaffold egress can be differentiated to the reticulocyte stage

**Figure 6:**
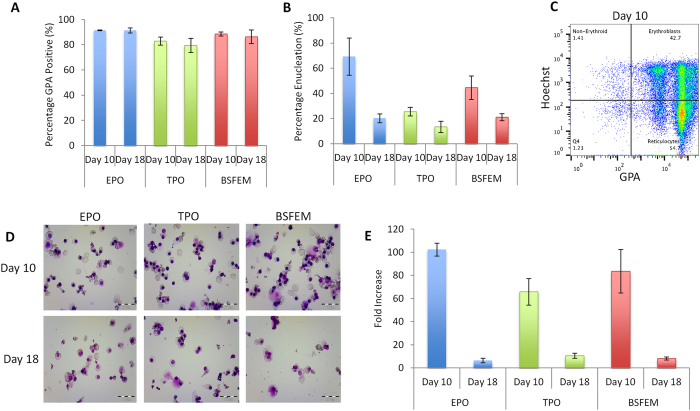
Early progenitors that egress from scaffold cultures differentiate to reticulocytes. Scaffold egress from days 10 and 18 was frozen and then defrosted and cultured in a standard erythroid expansion medium for 7 days and then in differentiation medium for a further 7 days to test their capacity to differentiate to reticulocytes. (**A**) GPA percentage positive cells at the endpoint (T168) of culture for each the BSFEM, +EPO and +TPO conditions. (**B,C**) Percentage enucleation at T168 as assessed using GPA and hoescht staining, a GPA^+^ and hoescht negative cell is considered to be a reticulocyte. This is also represented in the dot plot in (**C**). (**D**) Cells that are not enucleated reticulocytes are largely late orthochromatc erythroblasts as demonstrated by cytology analysis. Cells were cytospun and stained with May Grunwald Giemsa stains, images are representative and scale bars are 20 μm. (**E**) Expansion capacity of the starting material, expressed as fold increase from defrosting to the final stage of differentiation. N = 2 independent experiments, error bars represent the standard error of the mean.

To establish whether cells that egress from the scaffold have the potential to differentiate and enucleate to reticulocytes, egress populations from days 10 and 18 of culture were frozen and later cultured in standard 2D erythroid culture conditions. Thawed cells were first cultured in BSFEM with EPO as an expansion phase for 7 days and then terminally differentiated using high EPO, transferrin and insulin for a further 7 days. Importantly egress cells from all scaffold culture stages were successfully expanded and differentiated to the orthochromatic erythroblast or reticulocyte stage. GPA expression was observed on 90% of cells (Fig. 6A) with the appearance of reticulocytes confirmed using flow cytometry by the presence of DNA negative cells coupled with GPA expression (Fig. 6B,C) and cytology analysis (Fig. 6D). The remaining nucleated cells have a morphology largely consistent with orthochromatic erythroblasts (Fig. 6D).

Cellular progenitors from the egress population expand further post scaffold culture with a fold increase in cell number for day 10 egressed cells ranging from 65 fold for +TPO to 100 fold for +EPO; an 83 fold increase was noted for egress from BSFEM (Fig. 6E). There was however an increase in cell death and reduction in the expansion capacity for cells cultured from day 18, perhaps accounted for by the fact that cellular egress had been stored frozen or that the progenitors present may be more mature and so media conditions were not optimal for the later culture stages.

## Discussion

There is a need for improved approaches to the *ex vivo* manufacture of erythrocytes. Our aim was to mimic the structure of the bone marrow by compartmentalising and therefore restricting the differentiation of CD34^+^ cells in the honeycomb structure of the scaffold. We directly compared the efficacy of de-cellularised human bone scaffold with a biocompatible and non-biodegradable PU scaffold for the production of erythroid progenitors. Unlike previous studies^48^,^49^ that attempted to reproduce the whole of erythropoiesis within the scaffold, we have taken an alternative approach to seed CD34^+^ cells on scaffolds and then monitor the release of differentiating cellular egress from the scaffold. A thorough characterisation of the PU scaffold culture system was performed, exploring the cell populations that egress and the retained population within the scaffold. Although imaging and flow cytometry has been used before to assess scaffold cultures^41^,^50^, to our knowledge it has rarely been used in combination and to the extent utilised here.

This study has demonstrated the maintenance of a CD34^+^ population within PU scaffolds in basic media in the presence or absence of key cytokines (EPO, TPO) via immunofluorescence analysis. As the scaffold is moved every other day to a new well this provides evidence that is supportive of continued cellular egress being generated in scaffold cultures until day 28, testament in itself that early progenitors are retained within the scaffold structure. Erythroid progenitors were released from scaffold cultures for 28 days, although addition of TPO provided maximal scaffold egress (Fig. 1E,F). The cells that exit the scaffold can be easily harvested and these cells are then replenished every two days. Hence a key benefit of the PU scaffold used here is that it retains a continual cumulative pool of cells with further expansion potential. We have also shown that it is possible to seed scaffolds with the lineage depleted cell population isolated from peripheral blood (Fig. 1C,D), with potential application for culturing patient cells, especially useful if only a single blood sample is available, as the cells could be harvested every few days and utilized over the 28 days, however this would need to be explored further in future studies.

By first exploring the surface marker expression in normal 2D erythroid and megakaryocyte culture systems, we have shown that CD36, CD61 and GPA can be used to provide a convenient means of identifying approximately 90% of the scaffold output (Fig. 3). The bulk of the scaffold egress population are CD36^+^ from day 8 of the culture period alongside low CD61 positivity that closely resembled the early stages of the erythroid/megakaryocyte cultures when assessed using the same flow cytometry panel (Figs 3 and 4). This cell surface profile fits with the known surface expression profile for the transition between CMP and MEP stage being CD34^+/−^, CD36^+^ and CD41^+ ^9,11,12,13,51. There is also evidence that CD61 is present on MEPs, as we have observed a limited but consistent CD34^+^/CD61^+^ population throughout our cultures (data not shown)^12^. This is consistent with the fact that, CD41 and CD61 form the GPIIb/IIIa glycoprotein complex^52^. In the presence of TPO the CD34^+^/CD36^+^ MEP population persisted for a prolonged period compared to +EPO and BSFEM conditions, likely explained by the known expansion effects of TPO on HSCs^23^,^24^.

In addition to the retention of a proportion of cells derived from the original CD34^+^ population cells and then the continued release of progenitors, a significant benefit of scaffolds for the prolonged culture observed here compared to control cultures was the lower degree of cell death in PU CD34^+^ seeded cultures in the culture conditions used. Interestingly, there was consistently higher cell death regardless of the presence of exogenous EPO or not (Fig. 2C), suggestive that the cellular egress is comprised largely of an EPO independent population. EPO dependence occurs at the proerythroblast stage of the erythroid lineage^26^, consistent with the identification of scaffold egress as early progenitors; it is also possible that the cells are releasing small amounts of EPO within the scaffolds^53^ enabling limited support for a population that had transitioned into EPO dependence. Cell surface marker expression profiles using flow cytometry across the BSFEM, EPO and TPO conditions were highly homologous giving a largely CD34^+/−^CD36^+^GPA^lo^CD61^+^ population, providing further evidence that mainly early erythroid progenitors form the egress population, expression profiles also closely resembled the early stages (days 1–5) of the standard 2D erythroid and megakaryocyte cultures assessed using the same flow cytometry panel (Figs 3 and 4).

Importantly the scaffold egress at the different stages of culture can be expanded further and also differentiated to the reticulocyte stage. These data demonstrate that immature progenitors that egress from scaffolds are poised to further expand and terminally differentiate into reticulocytes. Reduced expansion and enucleation rates for cells at the later time point of day 18 were observed. This could be because the later cells are at different stages of proliferation or maturation and therefore exhibit different cytokine requirements resulting in spontaneous differentiation or apoptosis. A small number of cells which remained (1–2 × 10^5^) from the day 26 time point after analysis were also cultured further in 2D however too few cells were viable for analysis; thus providing evidence that expansion and survival becomes noticeably reduced by this point of the culture or that the late progenitors that egress at this point are not cryogenically stable (data not shown). Reduced expansion of scaffold egressed cells from later stages of the culture may also be indicative that although it is still largely erythroid progenitors that form the egress population, the cultures nevertheless are gradually progressing and maturing. Interestingly different rates of enucleation were also observed when scaffolds were cultured in BSFEM alone or with the addition of EPO or TPO, again possibly an effect of the cytokines causing cells to be at different stages of differentiation and lineage commitment. This would result in reticulocytes being produced at differing time points, possibly explaining the variation between the conditions, for example cells in the presence of EPO may be at a later stage of differentiation explaining higher enucleation rates.

In summary, we have successfully described, optimised and characterised a simplified porous PU scaffold culture system that retains a progressively decreasing population of CD34^+^ cells or cells derived from the original seeded population but also allows continuous egress of erythroid progenitors for harvesting (however these do become progressively differentiated over time). This study therefore acts as a foundation for future work, for example these scaffolds have the potential added benefit of lineage flexibility due to the continuous production of early progenitors that have the potential for directed differentiation down specific lineages, but this will need exploring further in future studies. Functionalisation of either this scaffold material or another second-generation scaffold to form a biologically enhanced compartmentalised system would provide a means to increase biomimicry of the bone marrow microenvironment, thereby introducing the possibility of increasing expansion and therefore the yield. Finally once optimal scaffold composition is achieved there is scope to scale this technology up to the bioreactor level and include perfusion in order to increase the capacity of production and potentially raise yields of *ex vivo* generated erythrocytes.

## Materials and Methods

### Antibodies

CD36, CD61, GPA, CD34 (Miltenyi Biotec), CD14 (BioLegend), Bric 235 (CD44, IBGRL reagents), Birma K3 (CD34, IBGRL reagents), Bric 256 (GPA, IBGRL Reagents), CD42b (BioLegend), Macrophage L1 calprotectin (Abcam catalogue number: ab22506).

### Peripheral Blood Mononuclear Cell and CD34^+^ cell Isolation

Peripheral blood mononuclear cells (PBMNC) were isolated from platelet apheresis blood waste (NHSBT, Bristol, UK) from healthy donors with informed consent. Ethics approval for all experimental protocols was granted by Bristol Research Ethics Committee (REC 12/SW/0199), and all methods were carried out in accordance with approved guidelines. PBMNC separation was performed using Histopaque 1077 as described previously^28^,^54^. CD34^+^ and lineage depleted cells were isolated from PBMNCs using magnetic activated cell separation (MACS, Miltenyi Biotec) as previously described^28^,^55^,^56^.

### Scaffold Preparation

Polyurethane scaffolds were produced as described previously^57^. Briefly 3D scaffolds were fabricated from dioxin by thermally induced phase separation giving a biocompatible and non-biodegradable honeycomb structure. Scaffolds with a pore size of 100–250 μm and a porosity of 90–95% were then manually cut into approximately 0.175 cm^3^ scaffolds (0.5 cm length by 0.5 cm width by 0.7 cm height) by first freezing the foam in liquid nitrogen and cutting to size using a razor blade. Human bone scaffolds were obtained with informed consent for research and development use (NHSBT, Liverpool, UK). Ethics approval for all experimental protocols was granted by Bristol Research Ethics Committee (REC 12/SW/0199), and all methods were carried out in accordance with approved guidelines. These consisted of cancellous bone harvested from the knee joint of donors and cut using a saggital saw to the approximate size of polyurethane scaffolds. Scaffolds were decellularised to a level of 99.92% as previously described^58^, and gamma irradiated.

Scaffolds, both the PU and the bone, were prepared as previously described^46^,^57^. Briefly, scaffolds were transferred to 70% ethanol and then washed by immersion in phosphate buffered saline (PBS) for 20 minutes. Scaffolds were centrifuged at 2,000 rpm for 10 minutes before the removal of PBS and immersion in collagen type 1 solution at 1 mg/ml (Sigma) in PBS for 20 minutes with rotation at room temperature. Scaffolds were then centrifuged again to allow full dispersal of the collagen through the structure at 2,000 rpm for 20 minutes; washed in PBS and centrifuged at 1,5000 rpm for 10 minutes and then exposed to ultra violet (UV) radiation for 15 minutes. The PBS was replaced with 70% ethanol for 2 hours at room temperature with rotation. Finally the scaffolds were washed twice with PBS to remove residual ethanol and stored in StemSpan (Stem Cell Technologies) with 10% fetal calf serum (FCS, Gibco) and penicillin/streptomycin at 100 U/0.1 mg per mL of media respectively (Sigma), for at least 2 days to equilibrate at 37 °C with 5% CO_2_.

### Three Dimensional Scaffold Cultures

Scaffolds were dried of storage media using sterilized 3 MM Whatmann paper and equilibrated to 37 °C until cell seeding. 0.5 × 10^6^ CD34^+^ or lineage depleted cells were seeded statically in 30–50 μL of media, as described below, the scaffolds were then incubated at 37 °C with 5% CO_2_ for 2 hours; with media added as required to ensure scaffolds did not dry out. 1.5 mL of basic serum free expansion medium (BSFEM) culture media consisting of StemSpan supplemented with; penicillin/streptomycin at 100U/0.1 mg per mL of media respectively (Sigma), cholesterol-rich lipids 40 μg/mL (Sigma), stem cell factor 100 ng/mL (SCF, Miltenyi Biotec), Interleukin-3 1 ng/mL (IL-3, R&D Systems), insulin like growth factor-1 40 ng/mL (IGF-1, R&D Systems), dexamethasone 1 μM/mL (Dex, Sigma), with or without thrombopoietin 50 ng/mL (Miltenyi Biotec) and with or without erythropoietin 2 U/mL (Bristol Royal Infirmary) was gently added to each scaffold. Full medium changes were performed every 2 days by carefully transferring the scaffold between wells of media using tweezers by picking up the scaffold gently from a single corner for minimal disruption (see Supplemental Figure S6). Although we were very careful during this transfer process a small number of cells that egress may be from the scaffold during this transfer process. Scaffold egress was counted using the MACSQuant flow cytometer (Miltenyi Biotec). Cells were auto-labelled with propidium iodide (Miltenyi Biotec) at a final concentration of 1 μg/mL. Dead cells were excluded and a live cell count taken as a density per millilitre. For cell death analysis samples were only used where 20% or more of the total population was the population of interest.

For two dimensional controls 0.5 × 10^6^ CD34^+^ cells were seeded in 24 well plates and maintained in equivalent culture conditions as described above minus the scaffold. Controls were matched as closely as possible to scaffold conditions by removing from the culture the average egress from the corresponding scaffold cultures. For these cells and scaffold egress not taken for flow cytometry analysis cells were frozen in 50% fetal calf serum (FCS, Gibco), 40% PBS with 10% DMSO (Sigma) and stored in liquid nitrogen until required.

### Erythroid expansion post 3D culture

Frozen cells were thawed from liquid nitrogen and cultured as described previously for 7 days expansion and then 7 days of terminal differentiation of erythroid progenitors^54^,^59^,^60^.

### Erythroid Culture to test for surface marker expression

Isolated CD34^+^ cells were re-suspended in IMDM (Biochrom, Source Biosciences) with 10 μg/mL insulin (Sigma), 3 U/mL heparin (Sigma), 200 μg/mL Transferrin (Sanquin, The Netherlands), 0.01 g/mL BSA (Sigma) and 6.8 mM sodium bicarbonate (NaHCO_3_) at appropriate density supplemented with; penicillin/streptomycin at 100U/0.1 mg per mL of media respectively (Sigma-Aldrich), cholesterol-rich lipids 40 μg/mL (Sigma-Aldrich), stem cell factor 100 ng/mL (SCF, Miltenyi Biotec), Interleukin-3 1 ng/mL (IL-3, R&D Systems), insulin like growth factor-1 40 ng/mL (IGF-1, R&D Systems), Dexamethasone 1 μM/mL (dex, Sigma) and erythropoietin 2 U/mL (Bristol Royal Infirmary). As above without IL-3 for a further 4 days with addition of media where required. For differentiation cells were washed three times with PBS and reseeded in IMDM base medium supplemented with 10 U/mL EPO (Bristol Royal Infirmary), 10 mg/mL insulin, 3% human AB plasma (Sigma), 1 mM thyroid hormone (T3, Sigma), 40 ng/mL IGF-1 (R&D systems) and 0.5 mg/mL holotransferrin (Sanquin, The Netherlands).

### Megakaryocyte Culture to test for surface marker expression

CD34^+^ cells were seeded in 12 well plates at a density of 1 × 10^5^/mL in CellGro supplemented with; 100 ng/mL thrombopoietin (TPO, Miltenyi Biotec), 10 ng/mL interleukin-1beta (IL1-β, Miltenyi Biotec), penicillin/streptomycin at 100U/0.1 mg per mL of media respectively (Sigma). Cells were split and fed when required.

### Flow Cytometry Panel

Flow cytometry was performed using 1 × 10^5^ cells labeled with extracellular conjugated antibodies for 30 min at 4 °C. Data were collected using a MacsQuant flow cytometer (Miltenyi Biotec) and processed using FlowJo Version 10.0.7. The following antibodies were included into a multicolour flow cytometry panel; CD34 coupled to VioBlue, CD36 coupled to PE, CD14 coupled to FITC, GPA with APC and CD61 with APC-Vio770. Relevant IgG controls were used to remove background signal from each antibody.

### Histology and Immunofluorescence

Scaffolds were fixed in 4% paraformaldehyde for 15–20 hours and washed four times in PBS for 15 minutes. Scaffolds were paraffin embedded and sectioned using a Leica RM2125 Microtome into 10 μm sections floated onto polysine slides (VWR). Slides were first baked at 56 °C for approximately 5 hours and then overnight at 37 °C. Slides were de-waxed by immersion into Histo-Clear (National Diagnostics) for 30–45 minutes before rehydration through a series of graded ethanol. For immunofluorescence sections were washed once in PBS before blocking with PBS containing 4% BSA (PBSA) for 1 hour. Slides were washed 5 times with PBS before incubating with primary antibody (primary and control antibodies are as stated in figure legends) for 1 hour, again washed 5 times with PBS and incubated with Alexa 647 secondary antibody (Invitrogen) for 1 hour. Slides were washed 5 times in PBS and incubated in DAPI to identify nuclei for 5 minutes before a further 2 washes. All steps are carried out at room temperature. For coverslips cells were fixed onto slide using 1% paraformaldehyde for 5 minutes before permeabilisation in 0.05% Triton (in PBS) for 5 minutes at room temperature. Coverslips were then washed three times and blocked and stained with antibodies as above. Finally coverslips and slides were washed and mounted using mowiol (Calbiochem). Samples were imaged using a Leica SP5 confocal microscope using a 63x lens (N.A. 1.4) in the Wolfson Bioimaging facility, University of Bristol.

### Scanning Electron Microscopy

Scaffolds were cut in half and mounted on a chuck with the centre of the scaffold face up. The samples were coated using an Emitech K575X sputter coater with a gold/palladium target and viewed with the Quanta 400 FEI Scanning Electron Microscope (SEM) in the Wolfson Bioimaging facility, University of Bristol.

### Statistical Analysis

Where appropriate statistical analysis in the form of a student’s t test, used to determine the level of significance where the following indicates, *P ≤ 0.05, **P ≤ 0.01, ***P ≤ 0.001. N = 3 for PU scaffold experiments with n = 2–3 for internal replicates. N = 2 for bone donor scaffold experiments with n = 3 for internal replicates.

## Additional Information

**How to cite this article**: Severn, C. E. *et al*. Polyurethane scaffolds seeded with CD34^+^ cells maintain early stem cells whilst also facilitating prolonged egress of haematopoietic progenitors. *Sci. Rep.* **6**, 32149; doi: 10.1038/srep32149 (2016).

## Supplementary Material

Supplementary Information
