# Supplementary material for: Polyurethane scaffolds seeded with CD34+ cells maintain early stem cells whilst also facilitating prolonged egress of haematopoietic progenitors
Source: Sci Rep. 2016 Aug 30;6:32149. doi: 10.1038/srep32149 (PMC5004174; doi:10.1038/srep32149)
Supplement: Supplementary Information [file srep32149-s1.pdf]

Polyurethane scaffolds seeded with CD34^{+} cells maintain early stem cells whilst also facilitating prolonged egress of haematopoietic progenitors.

Charlotte E Severn[1,2], Hugo Macedo[3], Mark J Eagle[4], Paul Rooney[4], Athanasios Mantalaris[3] and Ashley M Toye[1,2*]

Supplemental Figure S1

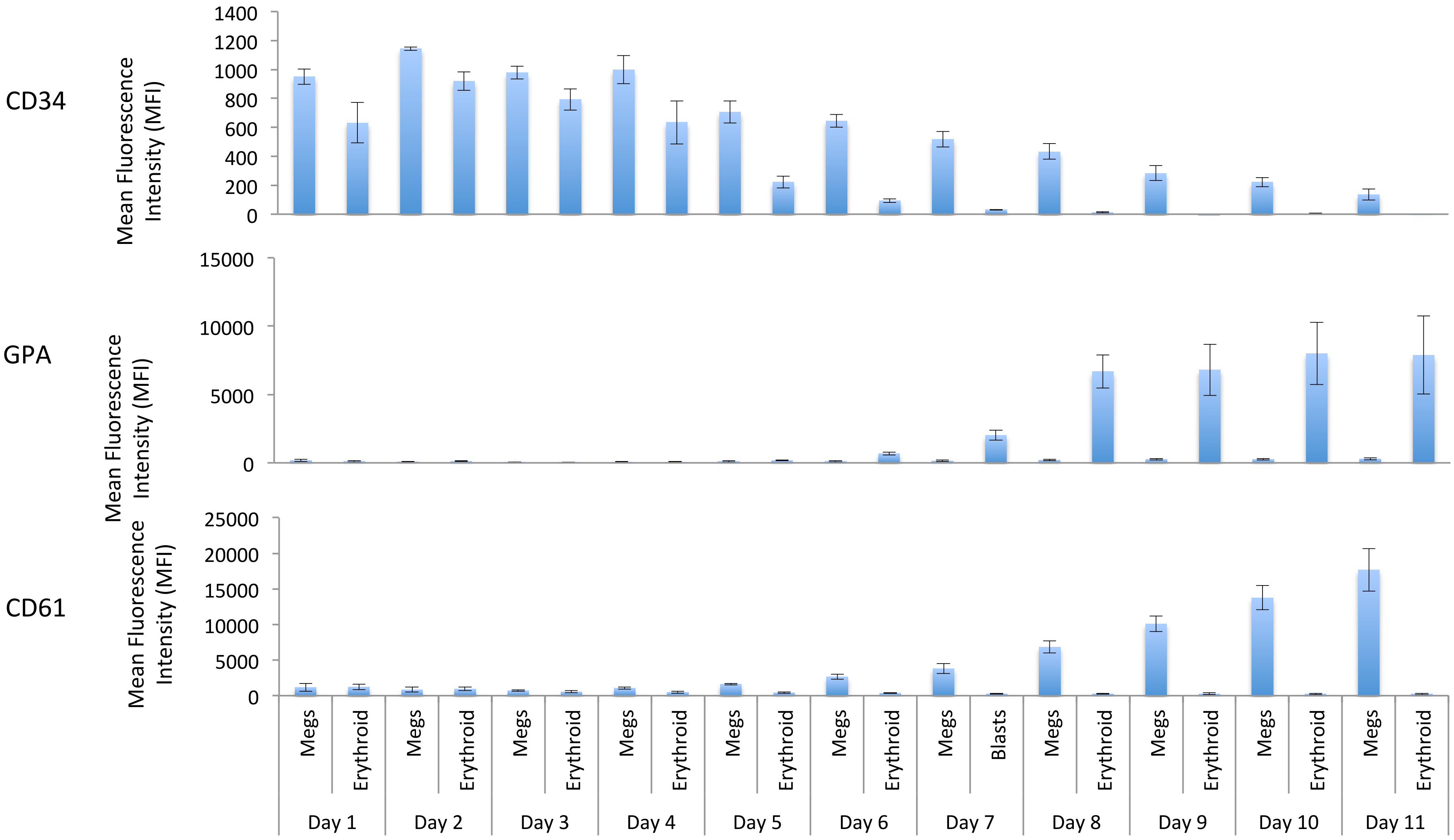

Figure S1: Analysis of cell surface marker density from erythroid and megakaryocyte cultures. Phenotype of erythroid and megakaryocyte cultures in respect to the density of CD34, CD61 and GPA on the cell surface, MFI for each cell surface marker taken from the total live cell population. CD34 (VioBlue, top), GPA (APC, middle) and CD61 (APC Vio770, bottom). N=3 independent experiments, error bars represent the standard error of the mean.

Supplemental Figure S2

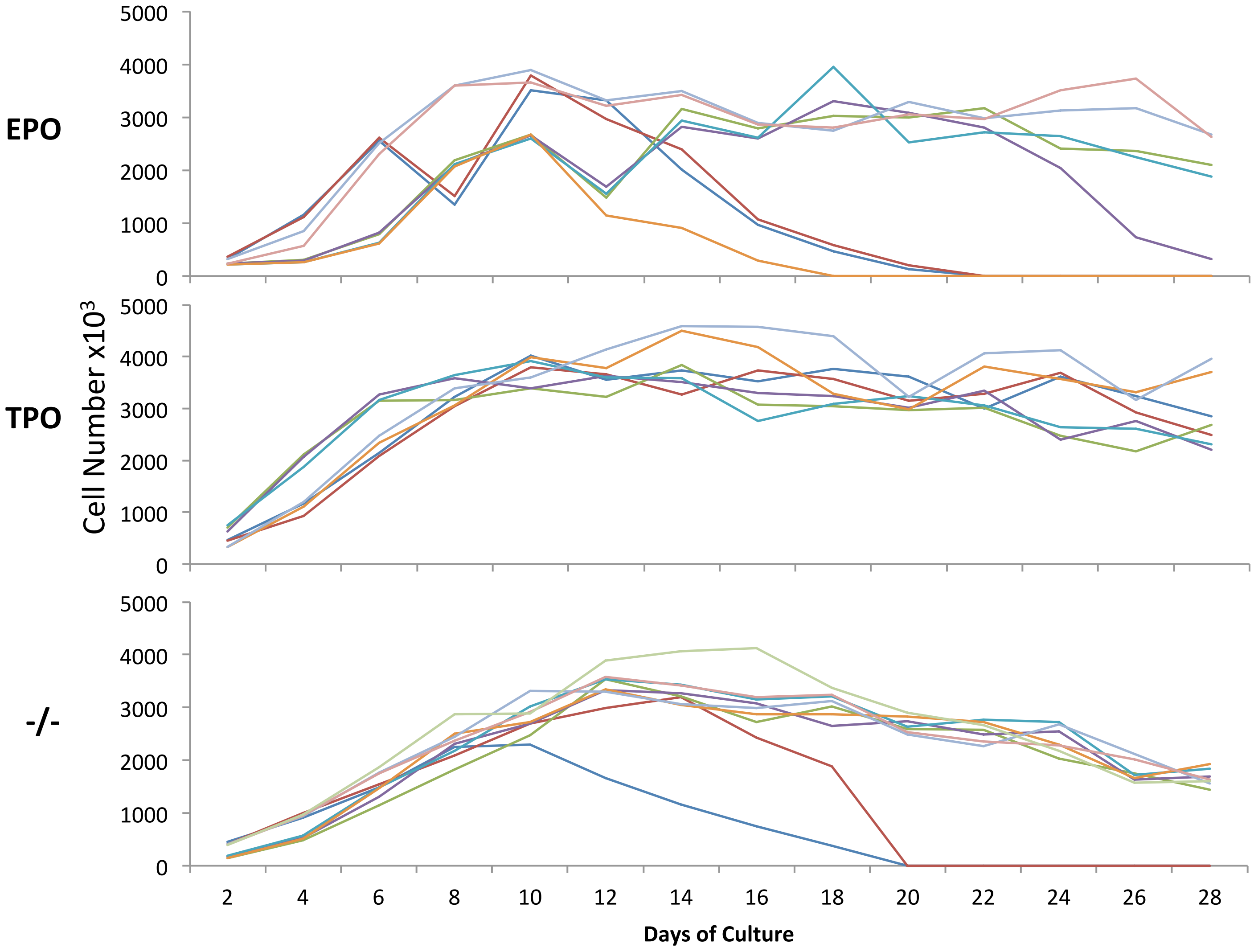

Figure S2: 2D high-density control cultures sustain cell growth for a 28 day culture period. High-density cultures consisted of 0.5×10^6 $CD34^+$ cells grown BFSEM, with or without the addition of EPO or TPO. The 2D cultures had a full media change every second day and were adjusted to mimic the scaffold egress process as closely as possibly by establishing the average cell egress of 3D cultures and removing this number of cells from each 2D control. N=3 experiments, in at least duplicate, all repeats from each experiment are shown. Trypan blue was used to remove the dead cells, cell counts were performed using the MACSQuant flow cytometer with a minimum of 30,000 events acquired.

Supplemental Figure S3

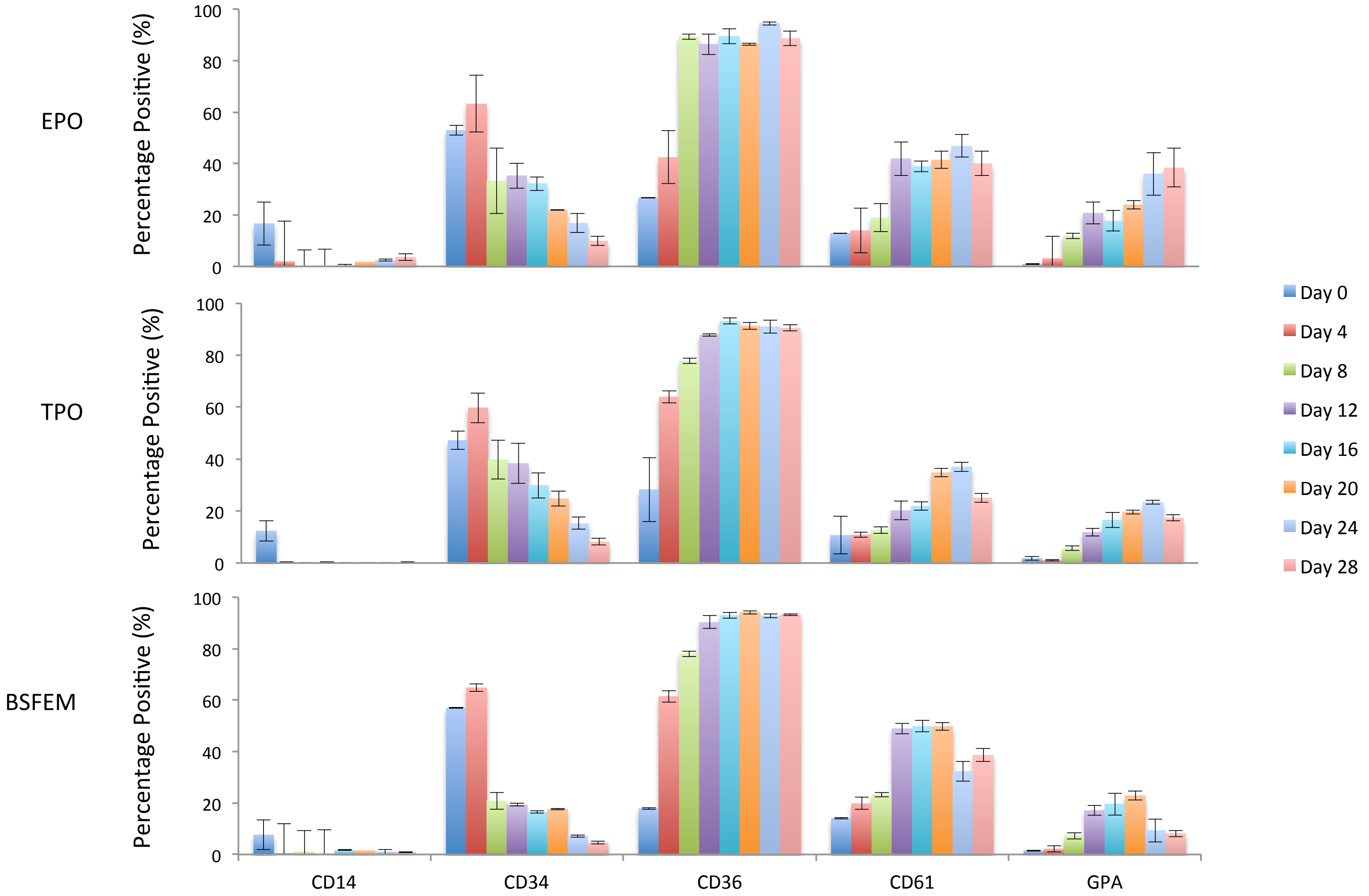

Figure S3: Flow cytometry analysis of control cell populations from cultures grown at high-density in the absence of a scaffold. Control cell populations were assessed every 4 days with antibodies to detect the following populations; CD14 (FITC), CD34 (VioBlue), CD36 (PE), CD61 (APC-Vio770) and GPA (APC). Cellular populations are shown as a percentage of the total live cell population for each condition, BSFEM alone or plus EPO, plus TPO. N=3 independent experiments in at least duplicate, error bars represent the standard error of the mean.

Supplemental Figure S4

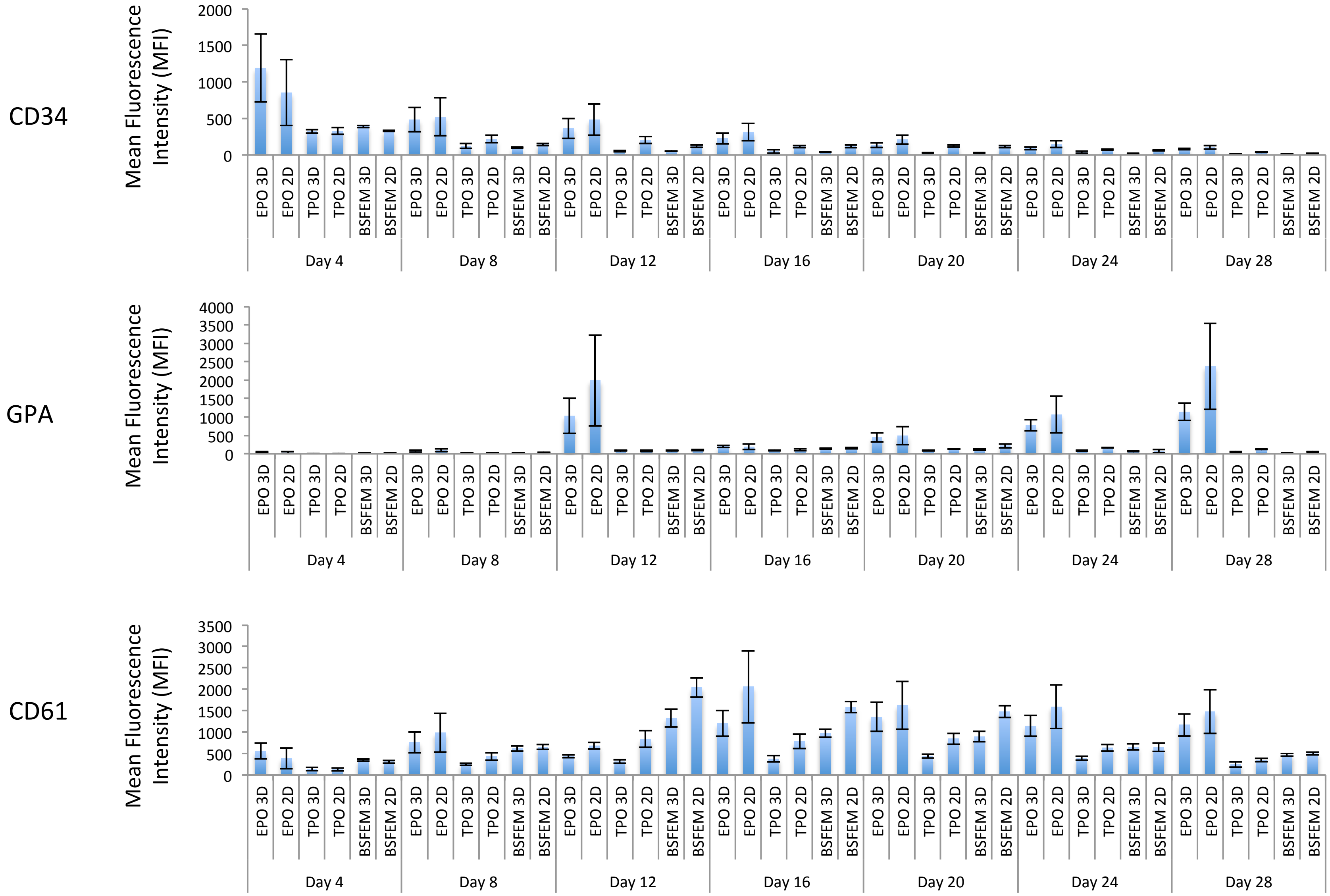

Figure S4: Density of surface expression from scaffold egress expressed as Mean Fluorescence Intensity (MFI) for CD34, GPA and CD61 expressing cells. Phenotype of scaffold egress in respect to density of CD34, CD61 and GPA on the cell surface. MFI for each cell surface marker was taken from the total live cell population for each condition BSFEM alone or plus EPO, plus TPO. N=3 independent experiments in at least duplicate, error bars represent the standard error of the mean.

Supplemental Figure S5

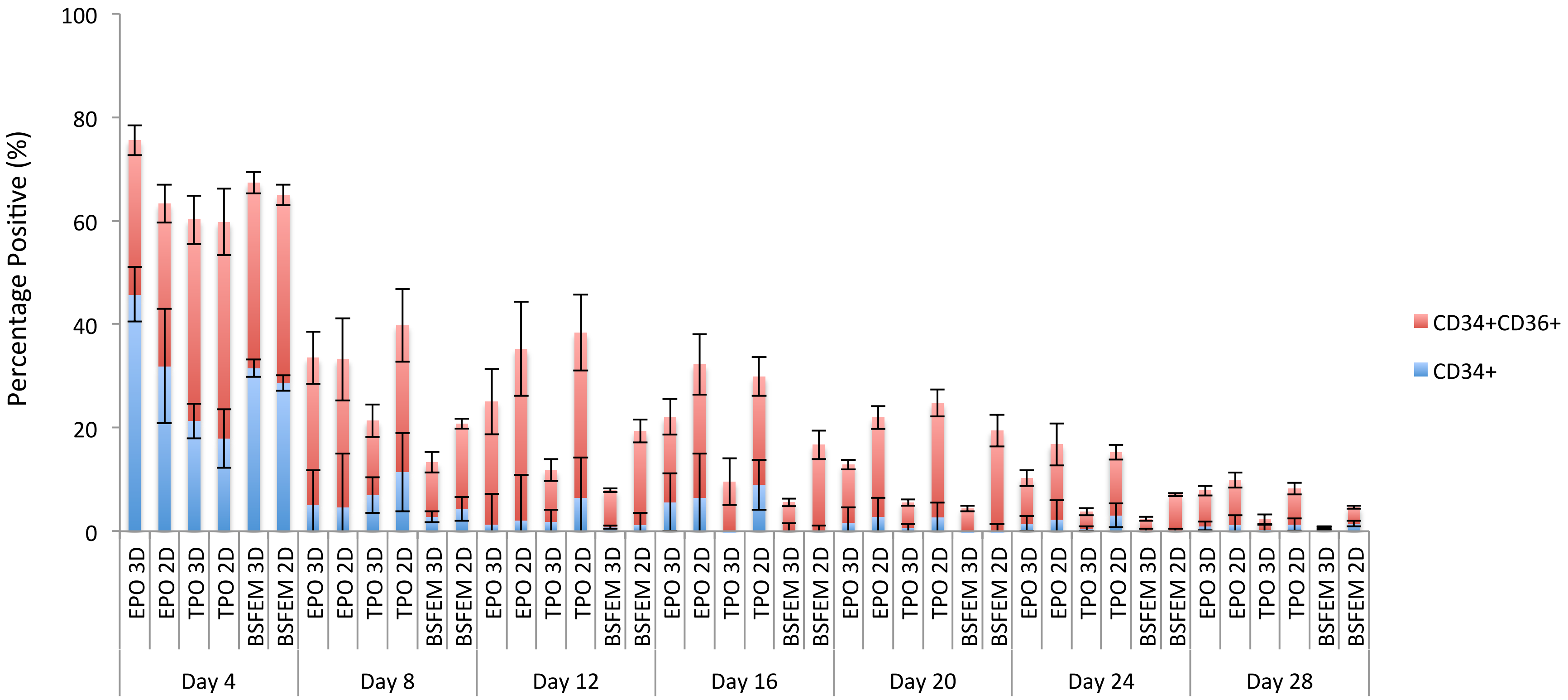

Figure S5: Comparison of CMP (CD34⁺/CD36⁻) and the MEP (CD34⁺/CD36⁺) cell populations between 3D and 2D cultures. Percentage of CMP and MEP populations as assessed using CD34 and CD36 as markers, expressed as a percentage of the total live population. Analysis every 4 days for each of the three conditions, EPO, TPO and BSFEM in 2D high-density control cultures and 3D scaffolds.N=3 independent experiments in at least duplicate, error bars represent the standard error of the mean.

Supplemental Figure S6

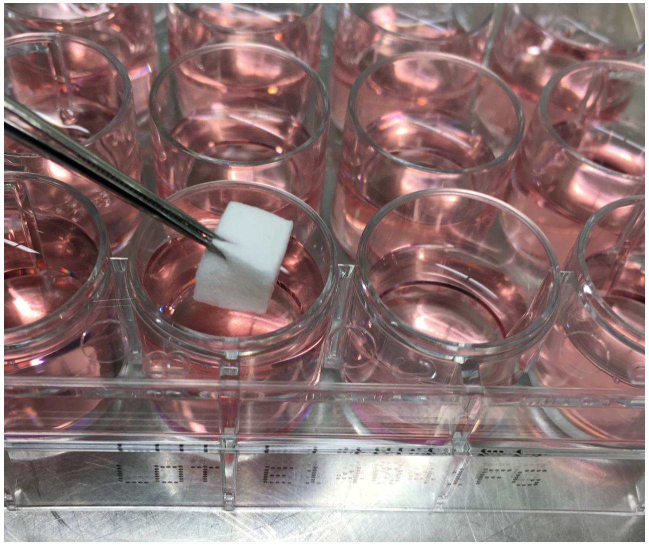

Figure S6: Method of transferring scaffolds during medium changes. Full medium changes are performed every 2 days by physically picking the scaffold up by the corner or edge with tweezers and transferring it to a new well with fresh medium.
